# Supplementary figures and images for: TREM2-Deficient Microglia Attenuate Tau Spreading In Vivo
Source: Cells. 2023 Jun 10;12(12):1597. doi: 10.3390/cells12121597 (PMC10296847; doi:10.3390/cells12121597)

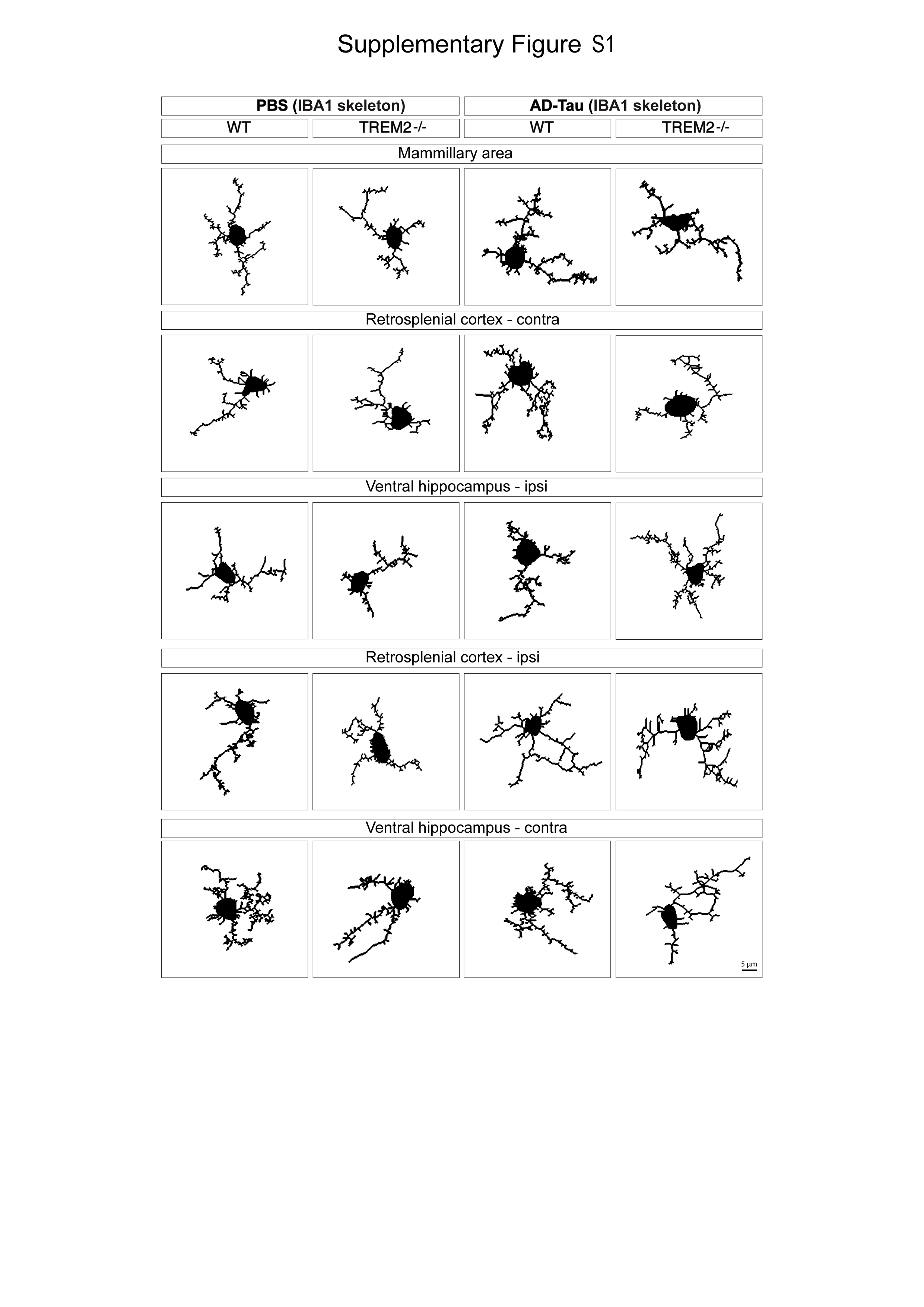

Supplement: Supplementary file 1 [file cells-12-01597-s001.zip › Supplementary_1.tif]

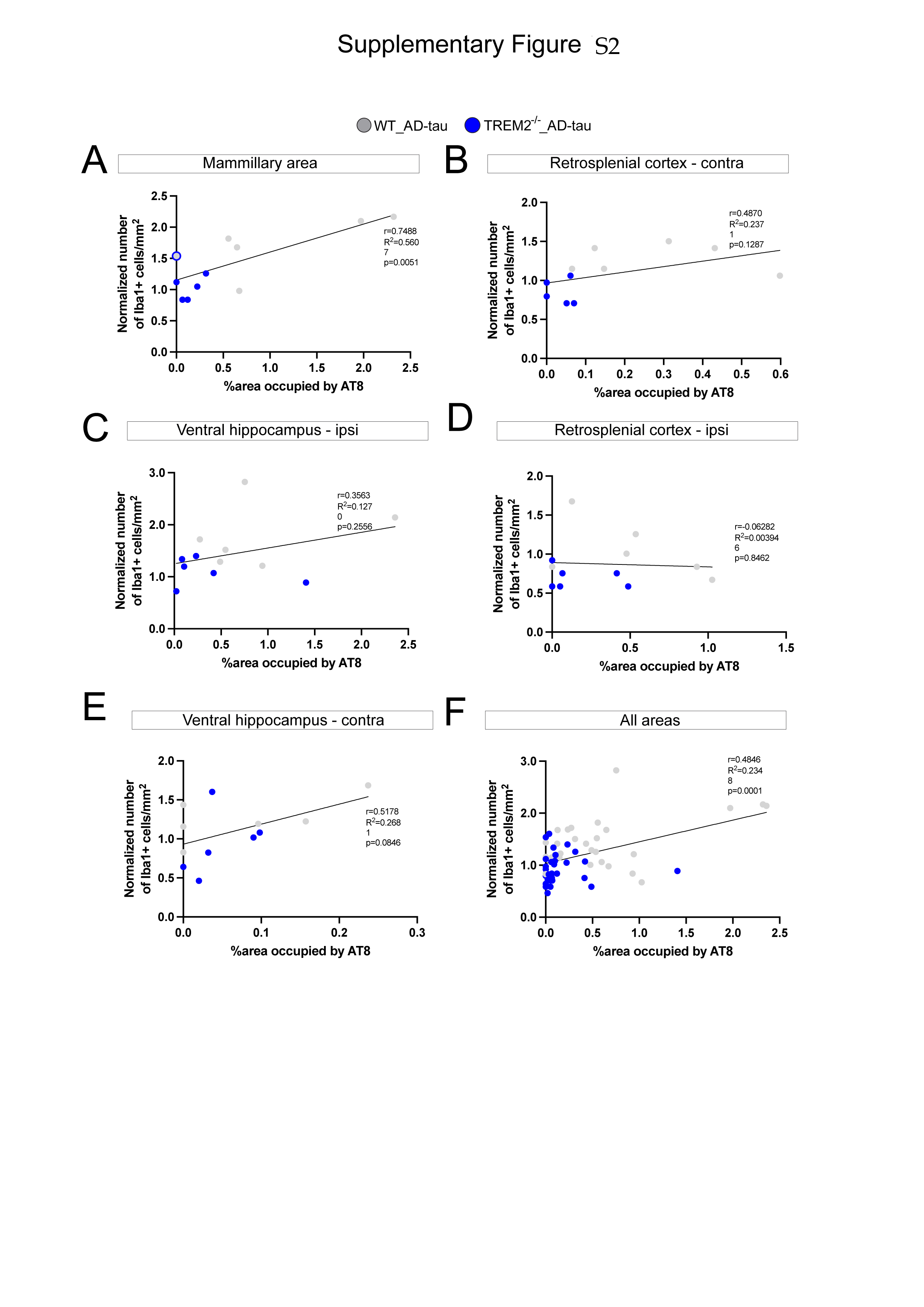

Supplement: Supplementary file 1 [file cells-12-01597-s001.zip › Supplementary_2.tif]

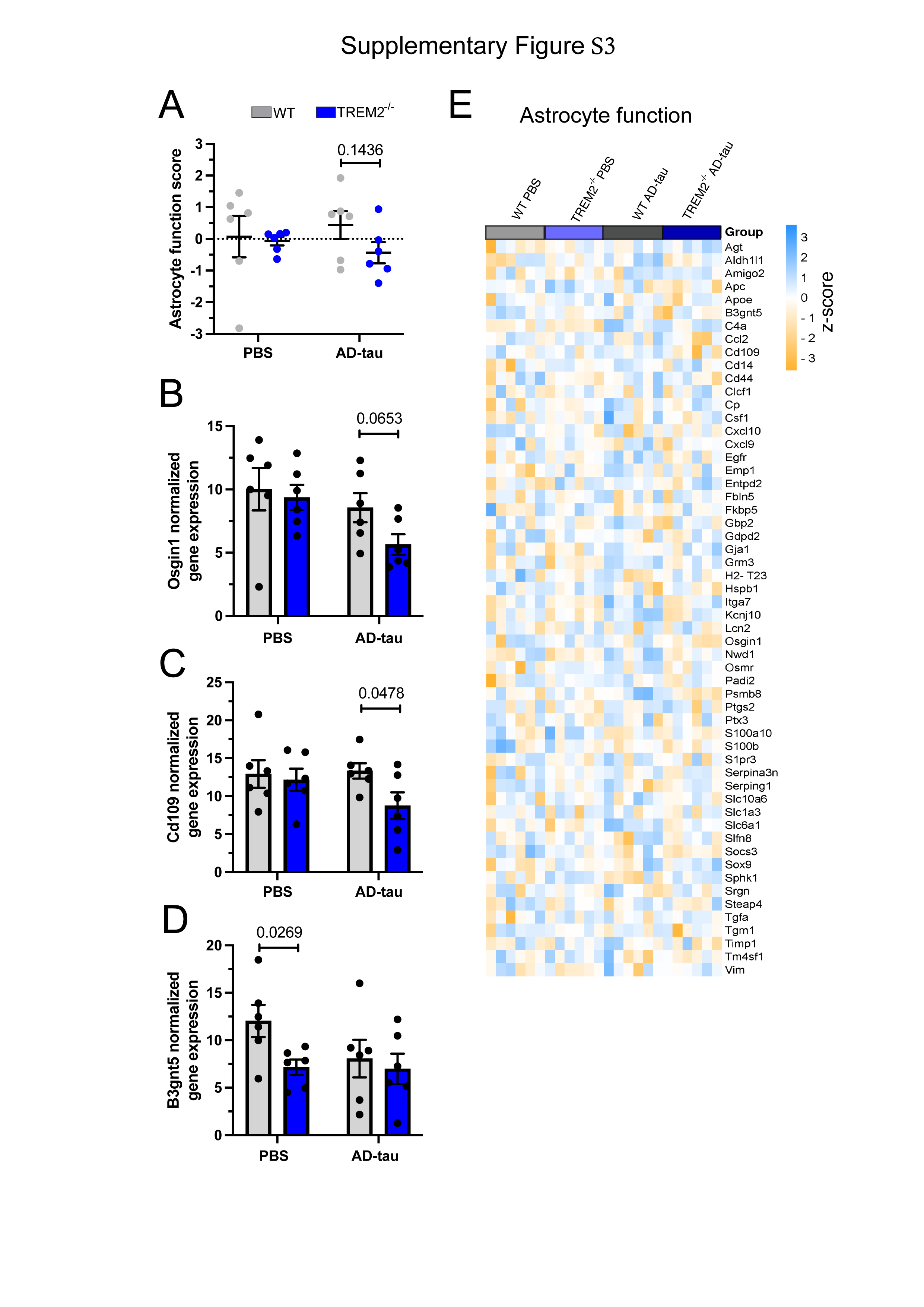

Supplement: Supplementary file 1 [file cells-12-01597-s001.zip › Supplementary_3.tif]
